# Endoscopic full-thickness resection of type 1 gastric neuroendocrine tumor: step-by-step description of technique

**DOI:** 10.1055/a-2436-1224

**Published:** 2024-10-25

**Authors:** Rafael Sartori Balbinot, Diego Cadena, Bruno Costa Martins, Cesar Capel de Clemente Junior, Evandro Sobroza de Mello, Adriana Vaz Safatle-Ribeiro, Fauze Maluf-Filho

**Affiliations:** 1 Endoscopy Division, Instituto do Câncer do Estado de São Paulo, University of São Paulo, São Paulo, Brazil; 2 Department of Pathology, Instituto do Câncer do Estado de São Paulo, University of São Paulo, São Paulo, Brazil

A 47-year-old woman with autoimmune chronic gastritis and type 1 gastric neuroendocrine tumor (gNET) was referred for resection of a gastric subepithelial lesion (SEL). Esophagogastroduodenoscopy showed atrophic pangastritis and a 13-mm SEL located at the anterior wall that was evaluated by endoscopic ultrasound ( Fig. 1 , Fig. 2 ).

**Fig. 1:**
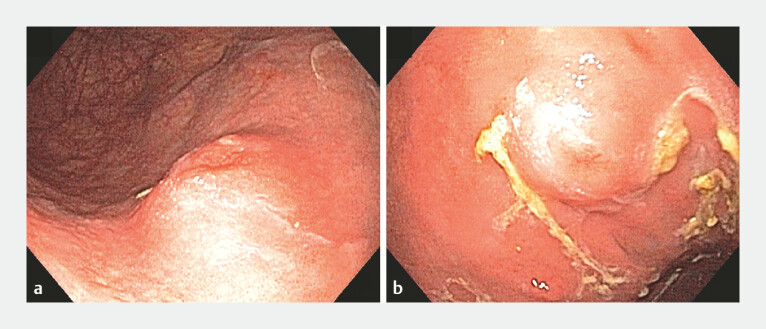
Endoscopic image of the subepithelial lesion located on the anterior wall of the proximal gastric body. **a** Lesion visualized in retroview. **b** Lesion visualized in direct view.

**Fig. 2:**
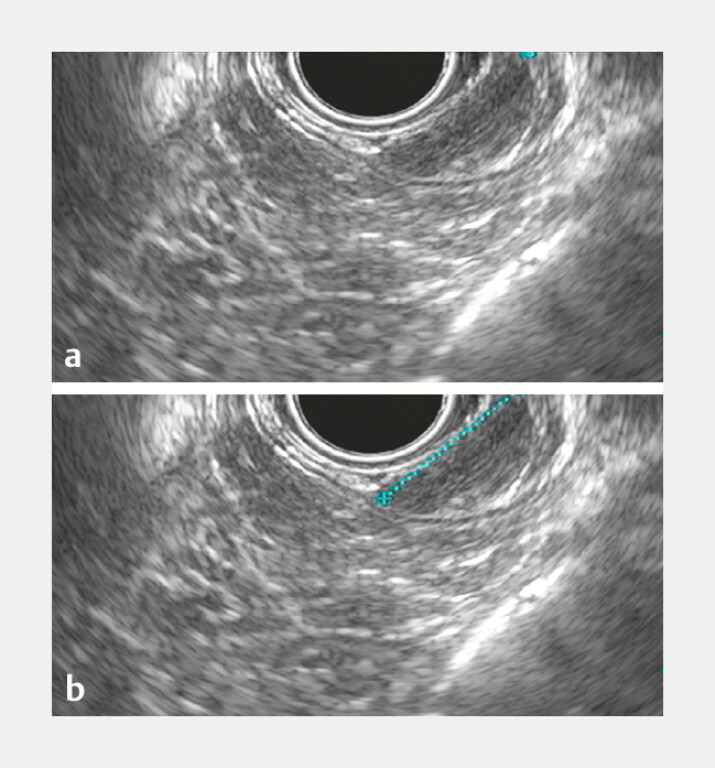
Endoscopic ultrasound (EUS) exposed an image with a hypoechoic echotexture, homogeneous, measuring approximately 13 mm, with precise limits, regular contours, and inserted in the submucosal layer. **a** EUS image showing the hypoechoic lesion. **b** EUS image with the marked lesion.

Endoscopic full-thickness resection (EFTR) was adopted. The technical description is demonstrated in Video 1 . The lesion was delimited with the marking probe. Then, another gastroscope previously mounted with the full-thickness resection device (FTRD) was used to perform the resection ( Fig. 3 ). The lesion was caught using the grasper. It was then necessary to apply suction to fully accommodate the lesion in the cap. Once the lesion was completely inside the cap, the clip was released by the handwheel, the snare was closed, and electrocautery was applied (VIO 300, AUTOCUT 100; Erbe Elektromedizin Gmbh, Tübingen, Germany). The patient had an uneventful recovery and was discharged on the first postoperative day. Histopathology confirmed a grade 2 well-differentiated neuroendocrine tumor involving the deepest third of the submucosa with free horizontal and vertical margins ( Fig. 4 ).

Endoscopic full-thickness resection of type 1 gastric neuroendocrine tumor using a full-thickness resection device. This method allows the resection of gastrointestinal lesions that cannot be resected using conventional techniques.Video 1

**Fig. 3:**
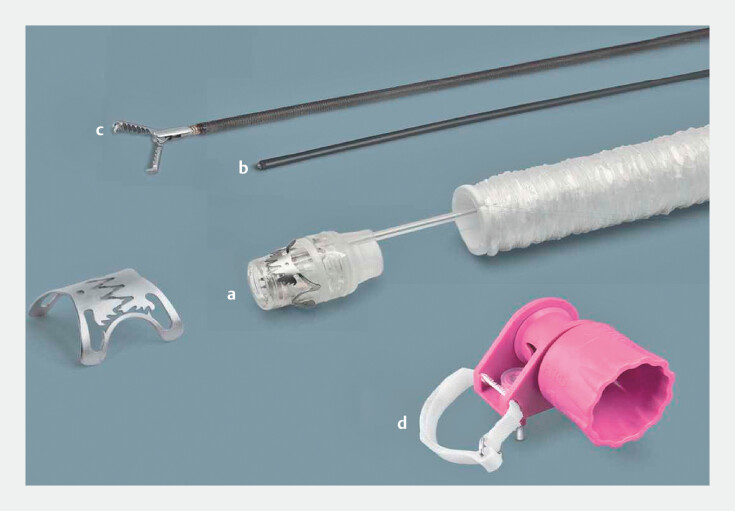
Device components of full-thickness resection device (FTRD). Two techniques were described for performing endoscopic full-thickness resection: exposed and non-exposed. The non-exposed technique can be performed using submucosal tunneling endoscopic resection (STER) or a specific device called an FTRD (Ovesco, Tübingen, Germany), which was used in the case. This article presents the resection using an FTRD device. **a** Cap loaded with an over-the-scope-clip and internal snare integrated. **b** Marking probe, a high frequency coagulation probe for marking of the target lesion in preparation of FTRD use. **c** Grasper, special grasping forceps to retrieve target tissue during an FTRD procedure. **d** FTRD handwheel.

**Fig. 4:**
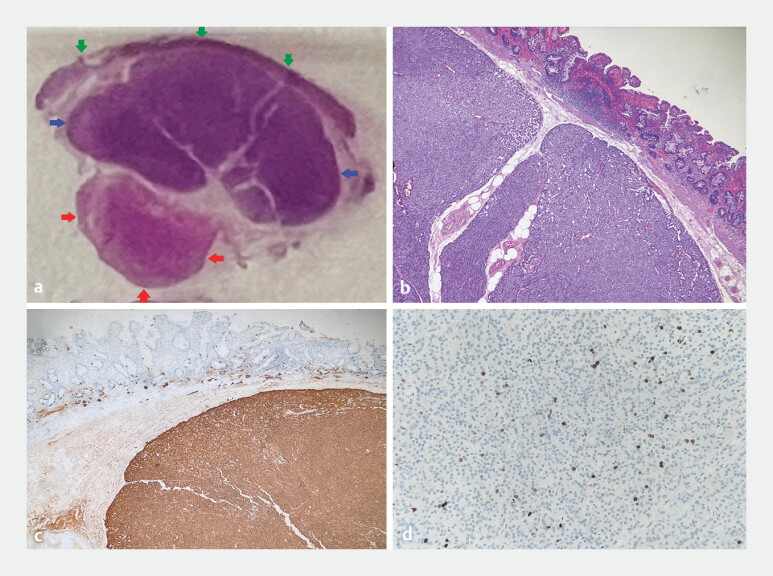
Histopathology and immunohistochemistry of grade 2 well-differentiated neuroendocrine tumor (gNET). **a** Green arrows point to the surface of gastric mucosa, blue arrows to the gNET limits, and red arrows to the muscularis propria margin. **b** Atrophic gastritis with intestinal metaplasia and gNET occupying the submucosa. **c** Intense positivity to chromogranin in neoplastic cells. **d** Ki-67 counting of 3.6% of the tumor cells.

EFTR is an emerging resection technique that allows resection of epithelial or subepithelial neoplastic lesions that affect the muscularis propria or are associated with fibrosis and not eligible for mucosectomy or submucosal resection 1 . EFTR appears to be effective for treating neuroendocrine tumors smaller than 10 mm 2 3 , although there may be a greater risk of incomplete resection for SELs measuring 15 mm. The American Gastroenterological Association and some current data suggest that EFTR be limited to lesions smaller than 15 mm 2 4 .

EFTR is a relatively new procedure that holds great potential for the resection of gastrointestinal wall lesions associated with fibrosis or that are embedded in deeper layers.

Endoscopy_UCTN_Code_TTT_1AO_2AG_3AF

## Notes

Endoscopy E-Videos https://eref.thieme.de/e-videos
*E-Videos* is an open access online section of the journal *Endoscopy* , reporting on interesting cases and new techniques in gastroenterological endoscopy. All papers include a high-quality video and are published with a Creative Commons CC-BY license. Endoscopy E-Videos qualify for HINARI discounts and waivers and eligibility is automatically checked during the submission process. We grant 100% waivers to articles whose corresponding authors are based in Group A countries and 50% waivers to those who are based in Group B countries as classified by Research4Life (see: https://www.research4life.org/access/eligibility/ ). This section has its own submission website at https://mc.manuscriptcentral.com/e-videos .
